# The association between levonorgestrel intrauterine devices and female fertility compared to other contraceptive methods: A historical cohort study

**DOI:** 10.1002/ijgo.70365

**Published:** 2025-07-11

**Authors:** Mette Peters Michaelsen, Laura Cæcilie Nielsen, Michelle Poulsen, Regitze Gyldenholm Skals, Søren Lundbye‐Christensen, Ulrik Schiøler Kesmodel

**Affiliations:** ^1^ Department of Obstetrics and Gynecology Aalborg University Hospital Aalborg Denmark; ^2^ Department of Health Science and Technology Aalborg University Aalborg Denmark; ^3^ Research Data and Biostatistics Aalborg University Hospital Aalborg Denmark; ^4^ The Fertility Unit Aalborg University Hospital Aalborg Denmark; ^5^ Department of Clinical Medicine Aalborg University Aalborg Denmark

**Keywords:** contraception, fertility, fertilization in vitro, insemination, intrauterine devices, live birth

## Abstract

**Objective:**

This study aimed to investigate whether the use of levonorgestrel intrauterine devices (LNG‐IUD) is associated with an increased need for medically assisted reproductive (MAR) treatment compared to other contraceptive methods.

**Methods:**

This register‐based historical cohort study included 733 875 Danish women aged 18–42 years between 2000 and 2018. The primary outcome was initiation of MAR treatment in the public healthcare sector. The exposure was categorized as the use of either LNG‐IUD, copper (Cu)‐IUD, progesterone‐only pills (POPs) or combined oral contraceptive pills (OCPs) during the study period. Cox proportional hazard regression was used to assess the risk of initiation of MAR treatment after LNG‐IUD use relative to other contraceptive methods.

**Results:**

Adjusted analysis revealed that POPs and OCPs were associated with increased hazards of MAR treatment relative to LNG‐IUD with hazard ratios (HR) of 2.91 (95% confidence interval (CI): 1.81–4.68, *P* < 0.001) and 6.58 (95% CI: 4.32–10.02, *P* < 0.001), respectively, while no difference was observed for Cu‐IUD relative to LNG‐IUD (HR 1.17, 95% CI: 0.61–2.25, *P* = 0.644).

**Conclusion:**

Use of LNG‐IUD was not associated with an increased need to undergo MAR treatment compared to Cu‐IUD, POPs and OCPs. However, due to limitations of the current study more research is needed in order to determine whether the use of LNG‐IUD has a negative effect on female fertility.

## 1 INTRODUCTION

Hormonal contraception is used throughout the world to prevent undesired pregnancies and to postpone parenthood.^1^, ^2^ Long‐acting reversible contraceptives (LARC) are especially popular, presumably due to their high efficacy and low maintenance. One LARC method is the levonorgestrel intrauterine device (LNG‐IUD), which has been increasingly used in recent years.^3^, ^4^, ^5^ The LNG‐IUD prevents fertilization and implantation by releasing levonorgestrel locally resulting in suppression of endometrial growth and thickening of cervical mucus making it impenetrable for spermatozoa.^3^ As LNG‐IUD is a reversible contraceptive method, removal should result in return to baseline fertility. However, current literature is inconsistent.^3^, ^6^, ^7^ Factors known to affect fertility include age, social status, tobacco‐ and alcohol use.^8^, ^9^, ^10^ However, little is known about how previous use of LNG‐IUD affects fertility beyond the period where it is used for preventive purposes. It is necessary to conduct more research on LNG‐IUD as contraception to uncover the possibility of it affecting long‐term fertility as a potential unwanted side effect. Therefore, the objective of this study was to investigate whether the use of LNG‐IUD was associated with an increased need for medically assisted reproductive (MAR) treatment compared to copper (Cu)‐IUD, combined oral contraceptive pills (OCPs) and progesterone‐only pills (POPs).

## 2 MATERIALS AND METHODS

This historical cohort study was based on data from Danish national registers. The study was reported according to Strengthening the Reporting of Observational Studies in Epidemiology.^11^

### 2.1 Study population

The study population consisted of Danish women 18–42 years old between January 1, 2000 and December 31, 2018. Information on contraceptive history was obtained from 1995. Exclusion criteria included Asherman syndrome, transcervical resection of the endometrium, and septum resection (Table S1), no time at risk of initiating MAR treatment, women with missing information of interest and contraceptive use for <12 months to represent valid use. Further, women with no use of contraception were excluded, as time at risk of initiating MAR treatment could not be defined as for contraception where the start of time at risk was defined by termination of use.

### 2.2 Data sources

Data was obtained from the Danish Population Statistics Register (DPSR), the Danish National Patient Register (DNPR),^12^ the Danish Medical Birth Register (DMBR),^13^ the Danish Education Register (DER),^14^ and the Danish National Prescription Register (NPR).^15^ Data was linked using each individual's personal identification number (CPR).

### 2.3 Study variables

Exposure was method of contraception, obtained from DNPR and NPR and categorized into LNG‐IUD (reference), Cu‐IUD, OCPs or POPs. Only use of one and not other contraceptive methods during the study period was included (restricted use), see below for details. The Danish Health Care Classification System codes and ATC codes used to define contraceptive use can be found in Table S2.

Primary outcome was initiation of MAR treatment in the public healthcare sector identified from DNPR and dichotomized as MAR treatment or no MAR treatment. During the study period, a criterion to receive public MAR treatment in Denmark included infertility in a couple trying for their first child together. Secondary outcome was live birth rate identified from DMBR and dichotomized as live birth or no live birth (including stillbirth).

Potential confounders were identified through directed acyclic graphs (Figure S1). These included age (18–22, >22–26, >26–30, >30–34, >34–38, >38–42 years, categorical), body mass index (BMI, calculated as weight in kilograms divided by the square of height in meters; 15–18.4, ≥18.5–24.9, ≥25.0–29.9, ≥30.0–34.9, ≥35.0–39.9, categorical), smoking (yes/no, categorical), educational level (low, middle, high according to the International Standard Classification of Education 2011, categorical), parity (0, 1, ≥2, categorical), duration of contraceptive use (1–3, >3–6, >6–9, >9–12, >12 years, categorical), and time at risk defined as previous time between contraceptive use (0–1, >1–2, >2–3, >3–4, >4–5, ≥5, years, categorical). Information on covariates was obtained from DNPR, NPR, DPSR, DMBR, and DER. BMI, parity, and smoking was only available for parous women. Information on age, educational level, parity, duration of contraceptive use, and time at risk was drawn at the end of the last period of contraceptive use (see below for details). Information on BMI and smoking was collected at the time of pregnancy.

### 2.4 Statistical analysis

Method and duration of contraceptive use was defined based on information from DNPR and NPR. When both IUD prescription/insertion and removal information was available, the exact length of use was used. If removal information was not available but a new insertion happened 5 years later, the IUD was assumed to have been used for the maximum recommended time for that IUD. If only prescription or insertion information was available, an estimated length of use of 4 years was used, based on scientific literature^16^, ^17^, ^18^ and knowledge on general use of IUDs in Denmark.^19^ When only an unspecific insertion or removal code was available, the IUD was assumed to be a Cu‐IUD. Length of use of oral contraceptives was identified based on prescriptions, and a cessation in use was defined as ≥4 weeks.

Contraceptive use and time at risk was assessed for each woman from inclusion or from their 18th birthday and until initiation of MAR treatment, until they turned 42, or until the end of the inclusion period, whichever occurred first. The age of 42 years was chosen as Danish legislation allows public MAR treatments up to the age of 41, thereby allowing time to experience pregnancy and live birth. A woman was considered at risk of initiating MAR treatment whenever a period of contraceptive use ended. If the woman started using the same contraceptive method again, a new time at risk would begin when the new period on contraception ended. Women were censored if they changed to another type of contraception or if an exclusion code occurred during the period. If birth occurred, women were censored 40 weeks prior to‐ and 8 weeks after birth and could thereafter enter the study again. One day was extracted from the last period of contraceptive use as some women used contraception until the day of MAR treatment initiation. All information was accumulated either as time using contraception, or time at risk. Analyses were performed based on the last entry for each woman. Complete case analysis was conducted.

Data was tested for normality using histograms. Normal distributed data are presented as means and standard deviations (SD), non‐normal distributed data as medians with 10th and 90th percentiles, and categorical data as frequencies and percentages.

A Cox proportional hazard regression was used to analyze the risk of initiating MAR treatment when using LNG‐IUD (reference) compared to other contraceptive methods. Assumptions of proportional hazards determined using a log–log plot of the survival and a test of zero slope of the Schoenfeld residuals were not fulfilled, and the analysis was therefore stratified upon the variables which showed violations. The final model was a Cox proportional hazards model analyzing the rate of initiating MAR treatment within 5 years from the last cessation of contraception stratified on age, duration of contraceptive use, previous risk time, and parity, and adjusted for educational level. A Cox proportional hazards regression model was used to analyze the risk of live birth. Individuals entered this analysis at or after 22 completed weeks of gestation when no longer at risk of miscarriage.^20^ Assumptions of proportional hazards were not fulfilled. Therefore, the analysis was stratified for age, duration of contraceptive use, previous risk time, parity, and educational level and thereby allowing for baseline hazards differing between strata. Analyses are presented as crude and adjusted hazard ratios (HRs) and 95% confidence intervals (95% CI).

BMI and smoking status were only available for parous women, as these variables are not routinely reported elsewhere. Therefore, to evaluate the impact of these potential confounders, logistic regression was used in a sensitivity analysis including BMI and smoking as covariates to assess the probability of having received MAR treatment in women who had given birth. Here, BMI <15 and >40 was excluded. This analysis was adjusted for age, duration of contraceptive use, educational level, and parity, as well as BMI and smoking. Results are presented as odds ratios (ORs) and 95% CI. A significance level of 0.05 was chosen for all analyses. Analyses were carried out using Stata 18 (StataCorp LLC).

### 2.5 Ethics

The study was registered with the North Denmark Region (November 22, 2022, ID: F2022‐136). In Denmark, register‐based research does not require an approval from the ethics committees nor patient consent. Data was handled in Statistics Denmark in accordance with Danish legislation. To gain access to NPR, an approval was obtained from the Danish Health Data Authority (December 9, 2022, FSEID‐00006398).

## 3 RESULTS

A total of 2 196 879 women were initially identified. After excluding women with no risk time, no contraceptive use during the study period, contraceptive use for <1 year, exclusion codes before study start, were ≥42 years at the time of risk start or had missing information, 733 875 women were included. In all exposure groups a medium level of education and being nulliparous was most prevalent. The total median age was 30.06, OCPs being the lowest at 29.94 and LNG‐IUD being the highest at 38.27 (Table 1).

**TABLE 1:** Characteristics of included women at their latest cessation of contraceptives.

	LNG‐IUD	Cu‐IUD	POPs	OCPs	Total	*P* value
*N* (%)	10 354 (1.4)	3821 (0.5)	9385 (1.3)	710 315 (96.8)	733 875 (100.0)	<0.001
Educational level, *n* (%)						
Low	2123 (20.5)	1210 (31.7)	2177 (23.2)	127 295 (17.9)	132 805 (18.1)	
Medium	4585 (44.3)	1698 (44.4)	4308 (45.9)	339 224 (47.8)	349 815 (47.7)	
High	3646 (35.2)	913 (23.9)	2900 (30.9)	243 796 (34.3)	251 255 (34.2)	<0.001
Parity, *n* (%)						
0	5003 (48.3)	2070 (54.2)	6163 (65.7)	353 570 (49.8)	366 806 (50.0)	
1	779 (7.5)	318 (8.3)	1057 (11.3)	144 995 (20.4)	147 149 (20.1)	
≥2	4572 (44.2)	1433 (37.5)	2165 (23.1)	211 750 (29.8)	219 920 (30.0)	<0.001
Age, years, median (10th–90th percentile)	38.27 (28.76; 41.39)	36.01 (25.67; 40.90)	30.34 (19.61; 39.75)	29.94 (21.35; 38.28)	30.06 (21.37; 38.51)	<0.001

Abbreviations: Cu‐IUD, copper intrauterine device; LNG‐IUD, levonorgestrel intrauterine device; OCP, combined oral contraceptive pill; POP, progesterone‐only pill.

Characteristics of BMI and smoking of parous women showed that in all groups, a higher percentage of the women were non‐smokers. The total median BMI was 22.95 (Table 2).

**TABLE 2:** Characteristics of included women who had given birth.

	LNG‐IUD	Cu‐IUD	POPs	OCPs	Total	*P* value
*N* (%)	2484 (0.9)	814 (0.3)	2022 (0.7)	283 472 (98.2)	288 792 (100.0)	<0.001
Smoking, *n* (%)						
No	2142 (86.2)	665 (81.7)	1790 (88.5)	243 684 (86.0)	248 281 (86.0)	
Yes	342 (13.8)	149 (18.3)	232 (11.5)	39 788 (14.0)	40 511 (14.0)	<0.001
BMI, median (10th–90th percentile)	22.72 (19.26; 30.30)	22.70 (18.82; 29.65)	22.59 (19.19; 29.41)	22.95 (19.41; 30.11)	22.95 (19.38; 30.11)	<0.001

*Note*: BMI, calculated as weight in kilograms divided by the square of height in meters.

Abbreviations: Cu‐IUD, copper intrauterine device; LNG‐IUD, levonorgestrel intrauterine device; OCP, combined oral contraceptive pill; POP, progesterone‐only pill.

### 3.1 Association between method of contraception and initiation of medically assisted reproductive treatment

Adjusted analysis showed an increased risk of initiating MAR treatment after the use of POPs and OCPs relative to LNG‐IUD. No difference was seen in the Cu‐IUD group relative to LNG‐IUD (Table 3).

**TABLE 3:** Analysis assessing the association between method of contraception and initiation of MAR treatment.

	HR	95% CI	*P* value	HR^a^	95% CI^a^	*P* value^a^
LNG‐IUD	1.00	Reference	—	1.00	Reference	—
Cu‐IUD	1.77	(0.92–3.41)	0.089	1.17	(0.61–2.25)	0.644
POPs	5.96	(3.71–9.57)	<0.001	2.91	(1.81–4.68)	<0.001
OCPs	20.74	(13.65–31.50)	<0.001	6.58	(4.32–10.02)	<0.001

*Note*: Cox proportional hazard regression was used.

Abbreviations: 95% CI, 95% confidence interval; Cu‐IUD, copper intrauterine device; HR, hazard ratio; LNG‐IUD, levonorgestrel intrauterine device; MAR, medically assisted reproductive; OCP, combined oral contraceptive pill; POP, progesterone‐only pill.

^a^ Stratified on age, time of contraceptive use, previous risk time and parity, and adjusted for educational level.

### 3.2 Association between method of contraception and live birth

Adjusted analysis showed that women who used Cu‐IUD, POPs and OCPs were more likely to experience live birth relative to LNG‐IUD (Table 4).

**TABLE 4:** Analysis assessing the association between method of contraception and live birth.

	HR	95% CI	*P* value	HR^a^	95% CI^a^	*P* value^a^
LNG‐IUD	1.00	Reference		1.00	Reference	
Cu‐IUD	1.50	(1.36–1.66)	<0.001	1.14	(1.03–1.26)	0.011
POPs	2.15	(1.99–2.33)	<0.001	1.21	(1.12–1.31)	<0.001
OCPs	4.98	(4.67–5.31)	<0.001	1.64	(1.54–1.76)	<0.001

*Note*: Cox proportional hazard regression was used.

Abbreviations: 95% CI, 95% confidence interval; Cu‐IUD, copper intrauterine device; HR, hazard ratio; LNG‐IUD, levonorgestrel intrauterine device; OCP, combined oral contraceptive pill; POP, progesterone‐only pill.

^a^ Stratified on age, time of contraceptive use, previous risk time, parity, and educational level.

### 3.3 Sensitivity analysis

Adjusted sensitivity analyses with and without BMI and smoking as covariates showed comparable ORs in all exposure groups. Increased odds of MAR treatment initiation was found after OCP use relative to LNG‐IUD, while no difference was seen for Cu‐IUD and POPs (Table S3).

## 4 DISCUSSION

In this study, use of LNG‐IUD was not associated with an increased need to undergo MAR treatment compared to Cu‐IUD, POPs and OCPs.

To our knowledge, this study is the first to use initiation of MAR treatment as an outcome when investigating the impact of LNG‐IUDs on female fertility. Other studies have investigated the time of return to fertility after contraceptive use. Some have reported that the time to conceive following contraceptive termination is not affected,^7^ while others have reported a delay in return to fertility.^3^, ^6^

In this study, the use of LNG‐IUD was associated with a lower risk of initiating MAR treatment compared to POPs and OCPs. IUD users had a higher median age and a larger proportion with higher parity. Previously, IUDs were only marketed for parous women. Further, even though the safety in nulliparous women has been established, surveys show that some healthcare professionals still believe that IUDs cannot be used by nulliparae.^21^ This could mean that nulliparous women might not be sufficiently represented in this study. Although our analyses were adjusted for age and parity, it is possible that the previous indication of parity for IUDs has affected the results, as couples already having a child together may have used private fertility clinics due to the national criteria to receive public MAR treatment during the study period. However, this study also showed that previous use of LNG‐IUD was associated with a lower HR of live birth compared to OCPs, POPs and Cu‐IUD. Yet, it is important to acknowledge that the current study does not allow a differentiation of whether the lower HR of live birth among previous LNG‐IUD users was due to a lower desire to conceive, a reduced use of MAR treatment or an actual decreased fertility.

Initiation of MAR treatment was not categorized by treatment type (e.g., insemination, in vitro fertilization and intracytoplasmic sperm injection). It is unknown whether the current findings are applicable to the different MAR treatments, and future research should aim to explore potential differences between MAR treatment types.

The sensitivity analysis showed that adjusting for BMI and smoking did not alter the results. This analysis cannot be interpreted as representative of the study population, as it is hampered by selection bias, since the analyses were based on parous women only. However, the comparable results indicate that BMI and smoking are not strong confounders in this study, and hence likely did not have a significant impact on the remaining analyses.

The contraceptive method with the highest risk of initiating MAR treatment relative to LNG‐IUD was OCP use. This might correlate with the fact that OCPs are frequently used in diseases known to affect female fertility such as endometriosis and polycystic ovary syndrome. It is possible that excluding diseases known to affect fertility to a degree where it is often associated with the need for MAR treatment would have affected our results. Future studies should take this into account.

In this study, national registers were used as the data source allowing rather large sample sizes. As all variables included in the study represent relatively objective measures, the risk of subjective reporting is minimal. Additionally, only women that used one type of contraception during the study period were included, thereby avoiding conflated effects as different contraceptives may have different effects on female fertility.

This study also had some limitations. Information on IUD insertion or removal performed by general medical practitioners was not available. Though this was compensated for by including prescriptions, Cu‐IUD users might not be fully represented as the ATC code for Cu‐IUDs is not used in Denmark. Further, some variables may only be available at time points that are not relevant for the data analysis. Initially, we planned to include civil status (heterosexual couple/homosexual couple/no partner) as a possible confounder. However, as this variable is highly time dependent, it was not possible to include it in a meaningful way. Additionally, missing data can be difficult to handle. In this study, missing data was handled by complete case analysis, which may have introduced some selection bias. Left truncation may have occurred, introducing selection bias, and underestimating the time at risk from termination of contraceptive use to time to event. Lastly, women who had never used contraceptives were excluded. It was not possible to include this group in a meaningful way because a time at risk could not be defined in the same manner as for users of contraception. Matching references with individuals from other exposure groups based on age would possibly introduce left truncation. Further, as use of contraceptives is still increasing, this group would be substantially smaller than the other groups, introducing the risk of excluding individuals due to matching.

## 5 CONCLUSIONS

The use of LNG‐IUD was not associated with an increased need for MAR treatment compared to other contraceptive methods. However, we acknowledge that the present study had limitations. Due to the rise in popularity of LNG‐IUD as contraception combined with the fact that LNG‐IUDs previously were considered for parous women only, future studies should aim to include more nulliparous women when investigating LNG‐IUDs effect on fertility, as their fecundability is yet to be studied.

## AUTHOR CONTRIBUTIONS

MPM: Conceptualization; data curation; formal analysis; funding acquisition; methodology; project administration; writing—original draft; writing—review and editing. LCN: Conceptualization; data curation; formal analysis; funding acquisition; methodology; project administration; writing—review and editing. MP: Data curation; funding acquisition; methodology; writing—review and editing. RGS: Formal analysis; writing—review and editing. SLC: Formal analysis; writing—review and editing. USK: Conceptualization; data curation; funding acquisition; methodology; project administration; supervision; writing—review and editing.

## FUNDING INFORMATION

This work was supported by the Clinic Surgery and Cancer Treatment Research Fund, The North Denmark Region (grant no.: 2024–011863‐12).

## CONFLICT OF INTEREST STATEMENT

MPM, LCN, MP, RGS and SLC have nothing to disclose. USK has received funding from Gedeon Richter Nordic, Merck and IBSA for studies outside this work; honoraria for teaching from Tillotts Pharma AB and Merck; and travel support and conference expenses from Merck.

## Supporting information


Data S1.

## Data Availability

Research data are not shared.
