# Supplementary material for: The association between levonorgestrel intrauterine devices and female fertility compared to other contraceptive methods: A historical cohort study
Source: Int J Gynaecol Obstet. 2025 Jul 11;172(1):305–10. doi: 10.1002/ijgo.70365 (PMC12724036; doi:10.1002/ijgo.70365)
Supplement: Supplementary file 1 — Data S1. [file IJGO-172-305-s001.docx]

**Supplemental figures and tables**

***The association between levonorgestrel intrauterine devices and female fertility compared to other contraceptive methods: a historical cohort study***


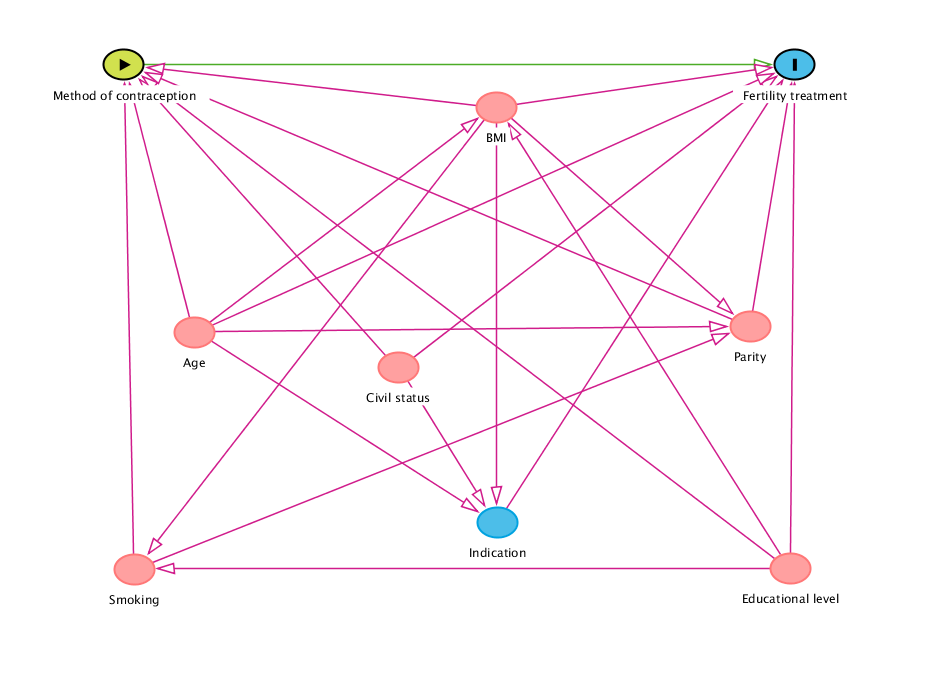


***Figure S1.*** *Directed acyclic graph showing potential confounding factors of the association between the use of contraceptives and the need for fertility treatment. Potential confounding factors included age, BMI (1-3), smoking (4,5), educational level (6-9), parity, and civil status. (Created with daggity.net, version 3.0)*

(1) Piirtola M, Jelenkovic A, Latvala A, Sund R, Honda C, Inui F, et al. Association of current and former smoking with body mass index: A study of smoking discordant twin pairs from 21 twin cohorts. PLoS ONE 2018 -07-12;13(7).

(2) Mosher WD, Lantos H, Burke AE. Obesity and contraceptive use among women 20–44years of age in the United States: results from the 2011–15 National Survey of Family Growth (NSFG). Contraception (Stoneham) 2018 May;97(5):392-398.

(3) Iversen DS, Kesmodel US, Ovesen PG. Associations between parity and maternal BMI in a population-based cohort study. Acta Obstet Gynecol Scand 2018 -03-01;97(6):694.

(4) Sequí-Canet JM, Sequí-Sabater JM, Marco-Sabater A, Corpas-Burgos F, Collar Del Castillo JI, Orta-Sibú N. Maternal factors associated with smoking during gestation and consequences in newborns: Results of an 18-year study. Journal of clinical and translational research 2022 Feb 25,;8(1):6-19.

(5) Rygh E, Gallefoss F, Grøtvedt L. Trends in maternal use of snus and smoking tobacco in pregnancy. A register study in southern Norway. BMC Pregnancy Childbirth 2019 -12;19(1).

(6) Ruokolainen O, Härkänen T, Lahti J, Haukkala A, Heliövaara M, Rahkonen O. Association between educational level and smoking cessation in an 11-year follow-up study of a national health survey. Scandinavian journal of public health 2021 Dec 1,;49(8):951-960.

(7) Lethbridge DJ. Use of contraceptives by women of upper socioeconomic status. Health care for women international 1990 Jan 01,;11(3):305-318.

(8) Goisis A, Håberg SE, Hanevik HI, Magnus MC, Kravdal Ø. The demographics of assisted reproductive technology births in a Nordic country. Human reproduction (Oxford) 2020 Jun 01,;35(6):1441-1450.

(9) Doll H, Vessey M, Painter R. Return of fertility in nulliparous women after discontinuation of the intrauterine device: comparison with women discontinuing other methods of contraception. BJOG : an international journal of obstetrics and gynaecology 2001 Mar;108(3):304-314.

***Table S1.*** *List of exclusion codes.*

| ***Exclusion code*** | ***Name*** |
| --- | --- |
| KLCB28 | Transcervical endometrial resection |
| KLCA16 | Endometrial destruction |
| KLCB25 | Hysteroscopic excision of pathological tissue in the uterus |
| KLCB32 | Hysteroscopic endometrial destruction |
| KLCC05 | Hysteroscopic resection of the uterine wall |
| KLCB22 | Hysteroscopic resection of pathological tissue in the endometrium |
| KLCB98 | Other hysteroscopic excision of pathological tissue in the uterus |
| KLCW98 | Other hysteroscopic operation on uterus |
| KLCG02 | Hysteroscopic adherence removal |
| KLCG98 | Other hysteroscopic reconstruction on uterus |
| DN856 | Adhesions in uterus |

Exclusion codes are based on The Danish Health Care Classification System (SKS).

**Table S2.** The Danish Health Care Classification System (SKS) codes and ATC codes used to define method and duration of contraceptive use.

| ***Contraceptive method*** | ***SKS codes*** | ***ATC codes*** |
| --- | --- | --- |
| LNG-IUD | MG02BA03, BJCD01, BJCZ01, BJCZ1, BJCZ2 | G02BA03 |
| Cu-IUD | MG02BA02, BJCD00, BJCZ00, BJCZ1, BJCZ2 | - |
| OCP | BJCA0 | G03AA07, G03AA079, G03AA10, G03AA11, G03AA12, G03AA14, G03AA16, G03AA18, G03AB03, G03AB05, G03AB08 |
| POP | BJCA1 | G03AC01, G03AC09, G03AC10 |

LNG-IUD = levonorgestrel intrauterine-device, Cu-IUD = copper intrauterine device, OCP = combined oral contraceptive pill, POP = progesterone-only pill.

**Table S3.** Sensitivity analysis adjusting for BMI and smoking.

|  | **OR** | **95% CI** | **P-value** | **OR_a_** | **95% CI_a_** | **P-value _a_** | **OR_b_** | **95% CI_b_** | **P-value _b_** |
| --- | --- | --- | --- | --- | --- | --- | --- | --- | --- |
| **LNG-IUD** | 1.0 | *Reference* |  | 1.0 | *Reference* |  | 1.0 | *Reference* |  |
| **Cu-IUD** | 1.31 | (0.50-3.42) | 0.581 | 0.44 | (0.15-1.30) | 0.138 | 0.44 | (0.15-1.31) | 0.141 |
| **POPs** | 4.57 | (2.52-8.27) | <0.001 | 0.83 | (0.41-1.68) | 0.603 | 0.82 | (0.40-1.68) | 0.590 |
| **OCPs** | 11.31 | (6.69-19.13) | <0.001 | 2.45 | (1.30-4.62) | 0.005 | 2.42 | (1.28-4.57) | 0.006 |

Multiple logistic regression was used. OR =Odds ratio, 95% CI = 95% confidence interval, LNG-IUD = levonorgestrel intrauterine-device, Cu-IUD = copper intrauterine device, OCP = combined oral contraceptive pill, POP = progesterone-only pill. a: Adjusted for age, time of contraceptive use, parity, and educational level. b: Adjusted for age, time of contraceptive use, parity, educational level, BMI, and smoking.
